# Supplementary material for: Description of a Newly Isolated Blautia faecis Strain and Its Benefit in Mouse Models of Post-Influenza Secondary Enteric and Pulmonary Infections
Source: Nutrients. 2022 Apr 1;14(7):1478. doi: 10.3390/nu14071478 (PMC9003314; doi:10.3390/nu14071478)
Supplement: Supplementary file 1 [file nutrients-14-01478-s001.zip › nutrients-1600794-supplementary.pdf]

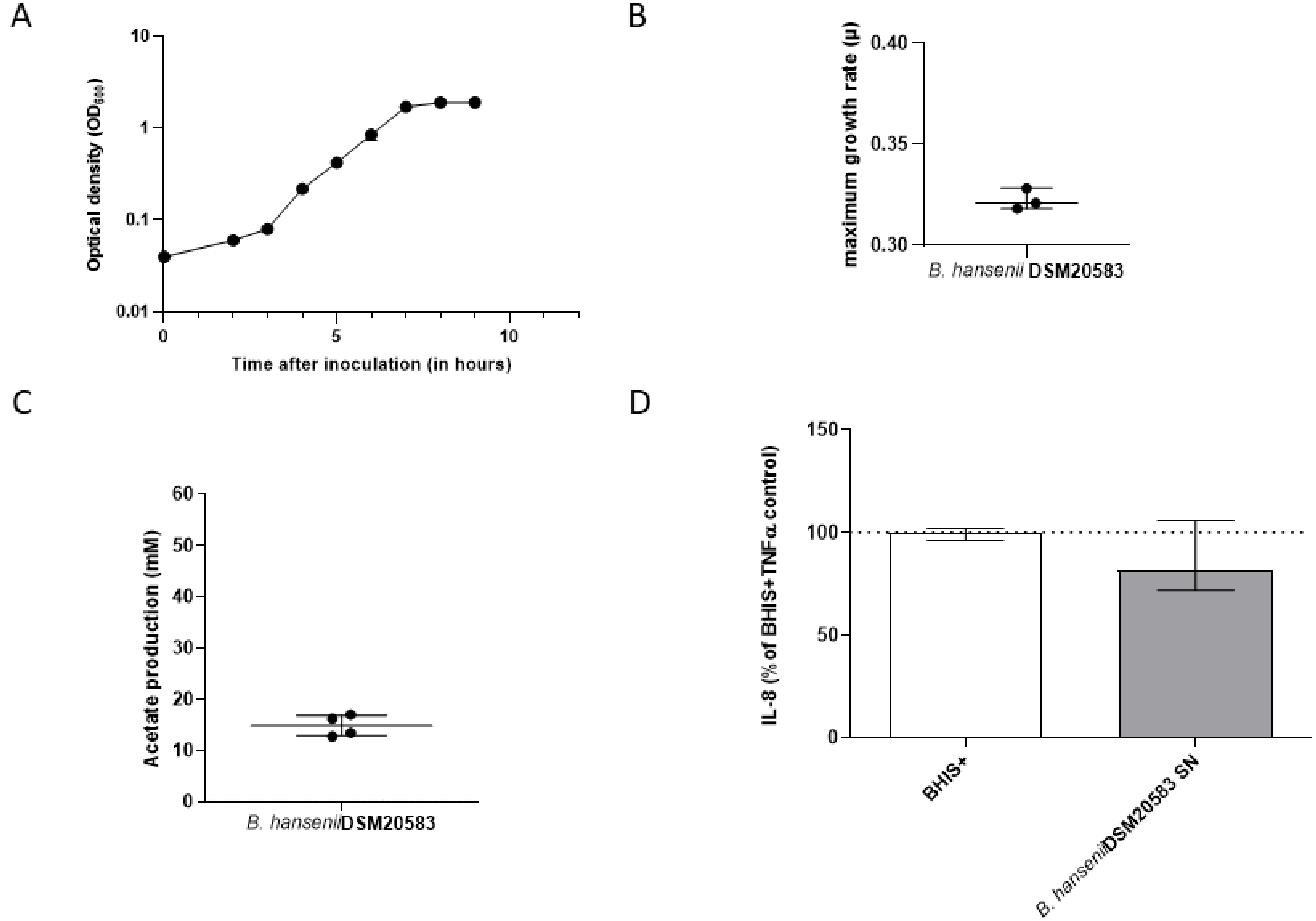

Figure S1 supplementary data : Growth characteristics of *B. hansenii* DSM20583 in BHIS+ medium and in vitro anti-inflammatory effect. A) Kinetic curve of *B. hansenii* DSM20583 grown in BHIS+. Logarithmic representation of the OD at 600 nanometers (nm), taken with one hour intervals. n=3. Median with interquartile range (IQR). B) Maximum growth rates (μ) of the growth curves. n=3. Median with interquartile range (IQR). C) Acetate production in mM by *B. hansenii* DSM20583, sampled in the stationary phase. Acetate levels of each sample were normalized with the acetate level of the BHIS+ medium without bacteria. n=4. Median with interquartile range (IQR). D) The IL-8 response of TNFα stimulated HT-29 cells supplemented with the culture supernatant (SN) (25%) of *B. hansenii* DSM20583 compared to the control with 25% BHIS+. n=6 Median with interquartile range (IQR). Significant when * $p<0.05$

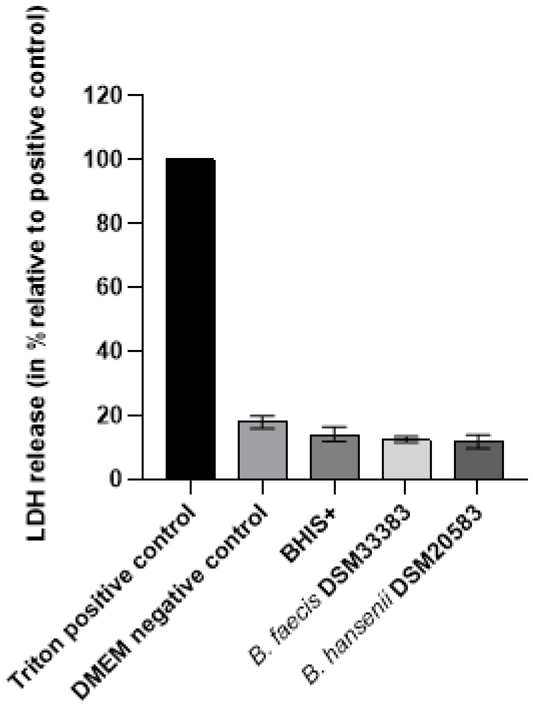

Figure S2 supplementary data : LDH release in DMEM supernatant from HT-29 cell cultures after 6 hours of co-incubation.
LDH release is expressed in % from the maximum LDH release, measured after administration of triton. LDH release in the cells grown in DMEM with TNFα was taken as negative control.

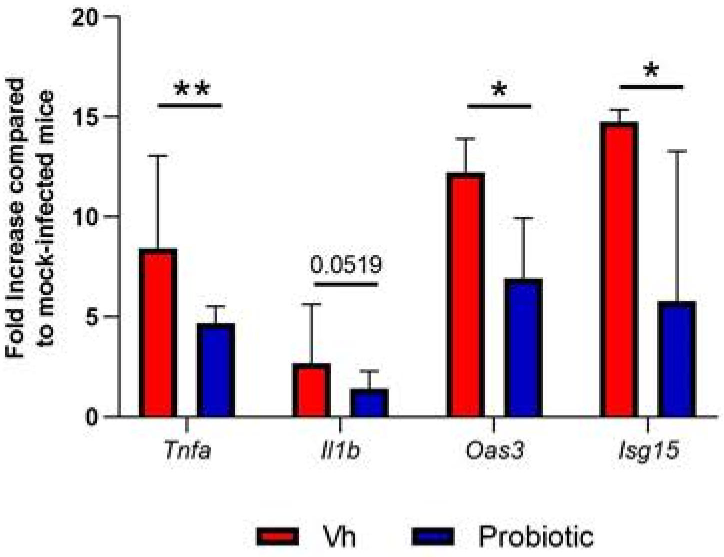

Figure S3 supplementary data. Impact of *B. faecis* DSM33383 administration on IAV infection. IAV-infected mice were gavaged or not with *B. faecis* DSM33383 ($5x10^6$ CFU/ 200µ1) at 2 d pi until 7 dpi. Gene expression in lung collected from infected mice at 8 d.p.i., either treated or not with *B. faecis* DSM33383 analyzed by RTqPCR. Significant differences were determined using the Mann-Whitney U test. (* P<0.05; ** P <0.01).

Table S1 supplementary data : 16S rDNA sequence of *Blautia faecis* DSM33383

**16S rDNA sequence *Blautia faecis* DSM33383**
actttatcagagagtttgatcctggctcaggatgaacgctggcggcgtgcttaacacatgcaagtcgaacgggaaatactttattgaaacttcggtggatttaatttatttctagtggcggacgggtgagtaacgcgtgg<br>gtaacctgccttatactgggggataacagccagaaatgactgctaataccgcataagcgcacagaaccgcatggttcggtgtgaaaaactccggtggtataagatggacccgcgttggattagctggttggcaggg<br>cagcggcctaccaaggcgacgatccatagccggcctgagagggtgaacggccacattgggactgagacacggcccagactcctacgggaggcagcagtggggaatattgcacaatgggggaaaccctgatg<br>cagcgacgccgcgtgaaggaagaagtatctcggtatgtaaacttctatcagcagggaagataatgacggtacctgactaagaagccccggctaactacgtgccagcagccgcggtaatacgtagggggcaagc<br>gttatccggatttactgggtgtaaagggagcgtagacggcgcagcaagtctgatgtgaaaggcaggggcttaacccctggactgcattggaaactgctgtgcttgagtgccggaggggtaagcggaattcctagtg<br>tagcggtgaaatgcgtagatattaggaggaacaccagtggcgaaggcggcttactggacggtaactgacgttgaggctcgaaagcgtggggagcaaacaggattagataccctggtagtccacgccgtaaacga<br>tgaatactaggtgtcagggagcacagctctttggtgccgccgcaaacgcattaagtattccacctggggagtacgttcgcaagaatgaaactcaaaggaattgacggggacccgcacaagcggtggagcatgtgg<br>tttaattcgaagcaacgcgaagaaccttaccaaatcttgacatccctctgaccgggacttaaccgtccctttccttcgggacaggggagacaggtggtgcatggttgtcgtcagctcgtgtcgtgagatgttgggttaa<br>gtcccgcaacgagcgcaacccctatccttagtagccagcacgtaatggtgggcactctgaggagactgccagggataacctggaggaaggcggggatgacgtcaaatcatcatgccccttatgatttgggctaca<br>cacgtgctacaatggcgtaaacaaagggaagcgaacctgtgagggtgggcaaatctcaaaaataacgtcccagttcggactgcagtctgcaactcgactgcacgaagctggaatcgctagtaatcgcggatcaga<br>atgccgcggtgaatacgttcccgggtcttgtacacaccgcccgtcacaccatgggagtcagtaacgcccgaagtcagtgacctaaccgcaagggaggagctgccgaaggcgggaccgatgactggggtgaagt<br>cgtaacaaggtagccgtatcggaaggtgcggctggatcacctcctttcta

Table S2 supplementary data : List of primers used for RT-PCR

**Primers**	
**Gapdh**	Forward 5'-GCAAAGTGGAGATTGTTGCCA-3'
	Reverse 5'-GCCTTGACTGTGCCGTTGA-3'
**Tnfα**	Forward 5'-CATCTTCTCAAAATTCGAGTGACAA-3',
	Reverse 5'-TGGGAGTAGACAAGGTACAACCC-3',
**Il1β**	Forward 5'-TCGTGCTGTCGGACCCATA-3',
	Reverse 5'-GTCGTTGCTTGGTTCTCCTTGT-3',
**Oas3**	Forward 5'-GTGGCACCGATGTCGAACTC-3'
	Reverse 5'-AGCAACATTCGCATGGCA-3'
**Isg15**	Forward 5'-GGCCACAGCAACATCTATGAGG-3'
	Reverse 5'-CTCGAAGCTCAGCCAGAACTG-3'

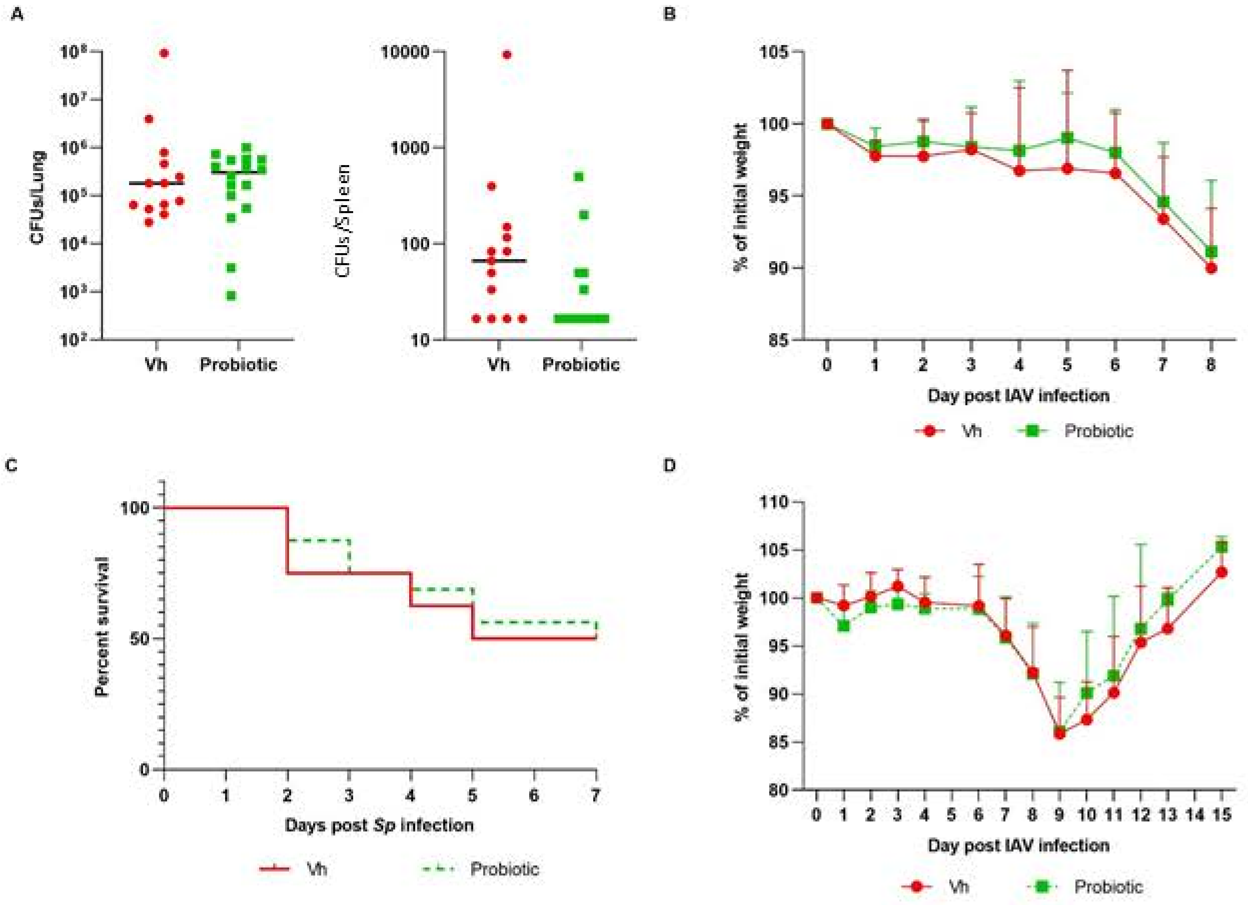

Figure S4 supplementary data. Effect of strain F2 (related to Coprococcus comes) supplementation during IAV infection on secondary *Streptococcus pneumoniae* infection. Schematic representation of the double infection system was shown in Figure 4. IAV-infected mice (8 d.p.i.) were infected with *S.*p ($1x10^6$ CFU). IAV-infected mice were treated or not with F2 (up to $2x10^7$ CFU/200µl) at 2 d.p.i. until 7 d.p.i. A) The number of bacteria was determined in lung (left) and spleen (right) 30h after the bacterial challenge (n=13-16, two pooled experiments shown). B) and D) the body weight evolution (in % initial body weight) and C) the survival of infected animals were monitored (n=16, two pooled experiments shown). No Significant differences were found using adapted tests.
